# Mild systemic inflammation enhances response to OnabotulinumtoxinA in chronic migraineurs

**DOI:** 10.1038/s41598-020-80283-4

**Published:** 2021-01-13

**Authors:** Yago Leira, Clara Domínguez, Pablo Ameijeira, Esteban López-Arias, Paulo Ávila-Gómez, María Pérez-Mato, Tomás Sobrino, Francisco Campos, Juan Blanco, Rogelio Leira

**Affiliations:** 1 Periodontology Unit, UCL Eastman Dental Institute and NIHR UCLH Biomedical Research Centre, University College London, 256 Gray’s Inn Road, London, WC1X 8LD UK; 2 Periodontology Unit, Faculty of Medicine and Odontology, University of Santiago de Compostela, Santiago de Compostela, Spain; 3 Medical-Surgical Dentistry (OMEQUI) Research Group, Health Research Institute of Santiago de Compostela (IDIS), Santiago de Compostela, Spain; 4 Clinical Neurosciences Research Laboratory, Health Research Institute of Santiago de Compostela (IDIS), Santiago de Compostela, Spain; 5 Department of Neurology, Headache Unit, University Clinical Hospital, University of Santiago de Compostela, Santiago de Compostela, Spain; 6 Neuroscience and Cerebrovascular Research Laboratory, Department of Neurology and Stroke Center, La Paz University Hospital, Neuroscience Area of IdiPAZ Health Research Institute, Universidad Autónoma de Madrid, Madrid, Spain

**Keywords:** Neurology, Neurological disorders, Headache

## Abstract

The anti-inflammatory effect of OnabotulinumtoxinA (OnabotA) has been a matter of discussion for many years. In chronic migraine, however, increased pro-inflammatory state is associated with good response to OnabotA. We aimed to investigate whether a mild systemic inflammatory state elicited by a common oral infection (periodontitis) could enhance treatment response to OnabotA. In this study, we included 61 chronic migraineurs otherwise healthy treated with OnabotA of which 7 were poor responders and 54 good responders. Before receiving OnabotA therapy, all participants underwent a full-mouth periodontal examination and blood samples were collected to determine serum levels of calcitonin gene-related peptide (CGRP), interleukin 6 (IL-6), IL-10 and high sensitivity C-reactive protein (hs-CRP). Periodontitis was present in 70.4% of responders and 28.6% of non-responders (P = 0.042). Responders showed greater levels of inflammation than non-responders (IL-6: 15.3 ± 8.7 vs. 9.2 ± 4.7 ng/mL, P = 0.016; CGRP: 18.8 ± 7.6 vs. 13.0 ± 3.1 pg/mL, P = 0.002; and hs-CRP: 3.9 ± 6.6 vs. 0.9 ± 0.8 mg/L, P = 0.003). A linear positive correlation was found between the amount of periodontal tissue inflamed in the oral cavity and markers of inflammation (IL-6: r = 0.270, P = 0.035; CGRP: r = 0.325, P = 0.011; and hs-CRP: r = 0.370, P = 0.003). This report shows that in presence of elevated systemic inflammatory markers related to periodontitis, OnabotA seems to reduce migraine attacks. The changes of scheduled inflammatory parameters after treatment and subsequent assessment during an adequate period still need to be done.

## Introduction

Periodontitis is a chronic infection affecting the gums that is characterized by gingival tissue breakdown, oral bone destruction and ultimately tooth loss. This oral infection does not only produce a local inflammatory reaction in the gingiva with upregulation of pro-inflammatory cytokines such as interleukin 6 (IL-6) and downregulation of anti-inflammatory cytokines such as IL-10 but also is considered an important contributor to the body’s overall inflammatory burden. It has been hypothesized that the ulcerated periodontal epithelium in periodontitis patients may act as an entrance to the bloodstream of IL-6 and many other pro-inflammatory molecules which evoke an acute-phase response in the liver resulting in the overexpression of systemic inflammatory mediators such as C-reactive protein (CRP), fibrinogen and serum amyloid A^1,2^ or even specific proteins involved in the process of neurogenic inflammation [i.e., calcitonin gene-related peptide (CGRP)] typically seen in the physiopathology of head pain^3^. Based on this, untreated periodontitis has been suggested to lead to a low-grade chronic systemic inflammatory state in both human and animal studies^4,5^.

Chronic migraine is a neurovascular disorder in which neuropeptides (e.g., CGRP, glutamate and substance P) and neurotransmitters are systemically released due to inadequate pain responses to peripheral chemical and mechanical stimuli^6^. Additionally, peripheral stimulation of meningeal nociceptors may also lead to the release of pro-inflammatory cytokines that activate mast cells and result in regional neuroinflammation^6^. OnabotulinumtoxinA (OnabotA) is the only prophylactic treatment specifically used for chronic migraine. It has been suggested that the mechanism of action of OnabotA is based on the blockage of inflammatory neuropeptides that are released from stimulated trigeminal sensory neurons^7^.

Experimental studies using different models of inflammation and pain yielded to contradictory results regarding the potential anti-inflammatory effect of OnabotA^8–11^. Bach-Rojecky et al. showed a lack of anti-inflammatory effect of peripheral application of OnabotA in two models of experimental neurogenic inflammation namely carrageenan and capsaicin^8^. On contrary, by using a formalin-induced inflammatory pain, Cui and co-workers were the first demonstrating a significant reduction in pain and oedema in the second phase of pain (inflammatory phase) but not in the first phase (acute pain phase) after OnabotA injections^9^. In line with these results, Chuang et al. showed that OnabotA pre-treatment dose dependently decreased inflammatory-cell accumulation and cyclooxygenase-2 expression in a capsaicin-induced prostatitis rat model^10^. Also, results from an organ culture study confirmed the ability of OnabotA to modify/reduce the expression of CGRP or cytokines in the trigeminal ganglion^11^. Recent clinical evidence has been published supporting these experimental findings, where Onabot A was capable of reducing CGRP levels in peripheral blood in chronic migraineurs^12^ and those with interictal pre-treatment increased concentrations of markers of neurogenic (CGRP)^13,14^ and systemic (acute-phase proteins such as pentraxin 3)^13^ inflammation responded better to this type of treatment.

It is reasonable to hypothesize, therefore, that mild systemic inflammation such as that observed in human periodontitis may enhance response to OnabotA in chronic migraineurs. To test this hypothesis, our aim was to evaluate whether those good responders to OnabotA presented high levels of inflammatory biomarkers compared to non-responders using a human model of low-grade systemic inflammation.

## Methods

### Study design

This is a secondary analysis with a cross-sectional design from previous observational studies from our group, which looked at the clinical and molecular relationship between periodontitis and chronic migraine^3,15^.

### Study population

For this analysis, we included sixty-one non-smoker adults in apparent good general health attending the Headache Unit of the University Clinical Hospital (Santiago de Compostela, Spain) with a diagnosis of chronic migraine (≥ 15 days per month with headache for at least 3 months)^16^ who were treated with pericranial OnabotA injections by a trained neurologist (RL) according to the Phase III REsearch Evaluating Migraine Prophylaxis Therapy (PREEMPT) protocol^17^. Briefly, chronic migraineurs received 155–195 OnabotA units in 31–39 injections sites twice over two consecutive periods of 12 weeks^13^. When present, treatment with other prophylactic medications was not interrupted. Evaluation of efficacy was done by means of diaries completed by patients in the 3 months following the second dose of OnabotA in which they had to report the number of episodes of moderate-severe acute headache lasting more than 4 h (or shorter if treated with symptomatic drugs). We considered responders those patients that showed ≥ 50% reduction in frequency of headache and non-responders were patients with < 50% of reduction in headache frequency^13^.

We excluded those participants aged 17 years or less, with 15 teeth or less (excluding third molars), who received periodontal treatment with or without systemic antibiotics in the last year, and who were pregnant or breastfeeding.

The study was approved by the Ethics Committee of the Servizo Galego de Saúde (ID: 2016/079) and performed according to the Declaration of Helsinki of the World Medical Association (2008). The Strengthening the Reporting of Observational Studies in Epidemiology (STROBE) guidelines were followed in this cross-sectional study^18^. Written informed consent was obtained from each participant after full explanation of the procedures.

### Migraine characteristics

Migraine characteristics including time evolution of chronic migraine (in months), frequency of migraine attacks (number/month), intensity of headaches (using the Visual Analogue Scale), presence of aura and allodynia were recorded.

### Socio-demographic, clinical and periodontal data

In addition to socio-demographic information (age, gender and education level) and body mass index (BMI: weight/height^2^), all participants received a full-mouth periodontal examination by a trained periodontist (PA) as previously described^15^. Full-mouth clinical periodontal measurements (i.e., six sites per tooth) from each participant including gingival pocket depth (PD), clinical attachment level (CAL), dental plaque accumulation, and gingival bleeding^19^ were obtained just before OnabotA treatment was initiated using a calibrated University of North Carolina periodontal probe (UNC15, Hu-Friedy, Chicago, IL, USA). The presence of periodontitis was established when ≥ 2 interproximal sites with CAL ≥ 3 mm and ≥ 2 interproximal sites with PD ≥ 4 mm (not on the same tooth) or 1 site with PD ≥ 5 mm were present^20^. Additionally, we calculated a measure of periodontitis activity, the periodontal inflamed surface area (PISA), which reflects the surface area of bleeding pocket epithelium in mm^2^^21^. PISA was calculated as follows: (1) with the mean CAL and gingival recession we obtained the periodontal epithelial surface area (PESA) for each tooth^22^; (2) the PESA value multiplied by the number of sites with bleeding upon probing results in the PISA for an specific tooth; (3) Full-mouth PISA is calculated for each participant (in mm^2^) by the sum of the PISAs for each tooth.

### Samples collection and laboratory methodology

Fasting blood samples were obtained in the morning in a pain free period (at least 12 h from the last migraine attack) and before initiating OnabotA therapy. Subjects had not consumed anti-inflammatory or analgesic medication in the previous 72 h. In brief, after blood samples were taken and clotted, serum was obtained by centrifugation (15 min at 3000*g*) and stored at − 80 °C. Serum high sensitivity CRP (hs-CRP) was measure using an immunodiagnostic IMMULITE 2000 Systems (Siemens Healthcare Diagnostics, Malvern, PA, USA) while IL-16 and IL-10 (BioLegend, San Diego, CA, USA) as well as CGRP (Cloud-Clone, Katy, TX, USA)] were measured by enzyme-linked immunosorbent assay technique following manufacturer instructions as previously described^3^. All biochemical determinations were done in an independent laboratory blinded to clinical data and treatment response.

### Statistical analysis

Mean values and standard deviation (mean ± SD) were calculated for continuous variables and compared using independent *t* test after normality was confirmed by Kolmogorov–Smirnov test. Non-normally distributed continuous variables were expressed as median [P_25_, P_75_] and compared with Mann–Whitney U test. Categorical data were reported as percentages (%) and compared by Fisher’s exact test. Parametric correlation analyses between clinical periodontal parameters and biomarkers among chronic migraine patients were performed using Pearson’s correlation coefficient. Logistic regression models were performed to test potential associations between periodontitis and response to treatment. All tests were performed at a significance level of α = 0.05. All data analyses were performed with IBM SPSS Statistics 20.0 software for Mac.

No formal sample size calculation was performed, as this is a secondary analysis. However, a post-hoc power analysis based on the results obtained from the present study and using our primary outcome (i.e., IL-6 concentrations) confirmed a 90% power to detect a 6.0 pg/mL difference in IL-6 between study groups (responders vs. non responders), with a SD of 2.1. Sufficient study statistical power (> 90%) was also confirmed when hs-CRP [effect (SD): 3.0 (0.9 mg/L)], IL-10 [effect (SD): 1.5 (0.4) pg/mL] or CGRP [effect (SD): 5.8 (1.6) pg/mL] were used for the calculation. All statistical power analysis were done with Macro !NSize for PASW Statistics (http://www.metodo.uab.cat/macros.htm).

## Results

### Baseline characteristics

Subjects with good (N = 54) and poor (N = 7) response were similar in terms of age (P = 0.609), gender (P = 0.885), low educational level (P = 0.594), BMI (P = 0.491), and migraine characteristics (number of migraine attacks/month: P = 0.411; intensity of migraine attacks: P = 0.264; presence of allodynia: P = 0.655; and presence of aura: P = 0.339). Only the time of chronic migraine evolution was statistically significant less in responders than non-responders (24.0 ± 14.2 vs. 34.5 ± 9.8 months, P = 0.032) (Table 1).

**Table 1:** Characteristics of chronic migraineurs according to treatment response (N = 61).

Variable	Responders (N = 54)	Non-responders (N = 7)	P value
Age (years)	49.0 ± 9.4	50.8 ± 3.3	0.609
Females, n (%)	53 (98.1)	7 (100)	0.885
Low education level, n (%)	25 (46.3)	3 (42.9)	0.594
BMI (kg/m^2^)	26.0 [24.7, 28.0]	24.0 [22.0, 27.2]	0.259
**Clinical periodontal parameters**			
FMPS (%)	38.0 ± 22.2	30.5 ± 23.6	0.453
FMBS (%)	54.1 ± 28.4	24.4 ± 12.4	**< 0.001**
Mean PD (mm)	3.2 ± 0.6	2.5 ± 0.7	**0.006**
PD6, n	12.3 ± 15.1	1.6 ± 2.4	**< 0.001**
Mean CAL (mm)	3.8 ± 0.9	2.9 ± 0.8	**0.033**
CAL5, n	33.6 ± 28.0	15.7 ± 10.8	**0.005**
Mean PISA (mm^2^)	630.0 ± 558.7	360.1 ± 214.0	**0.025**
**Migraine characteristics**			
Time of evolution (years)	24.0 ± 14.2	34.5 ± 9.8	**0.032**
Frequency (nº attacks/month)	19.6 ± 5.4	21.4 ± 3.9	0.411
Intensity (VAS)	8.5 [8.0, 10.0]	8.0 [7.0, 9.0]	0.250
Aura, n (%)	0 (0.0)	4 (7.4)	0.339
Allodynia, n (%)	34 (68.0)	5 (83.3)	0.655
Analgesic overuse, n (%)	13 (25.5)	2 (33.3)	0.648
**Preventive treatment in the last 3 months, n (%)**			
Topiramate	17 (32.1)	1 (14.3)	0.663
β-Blockers	20 (37.0)	4 (57.1)	0.418
Amitriptyline	22 (40.7)	4 (57.1)	0.409
Flunarizine	7 (13.0)	1 (814.3)	0.647
Antihypertensives	0 (0.0)	1 (14.3)	0.115
**Migraine acute treatment, n (%)**			
Triptans	42 (77.8)	5 (71.4)	0.655
Non-steroidal anti-inflammatory drugs	50 (92.6)	5 (71.4)	0.136
Opioids	13 (24.1)	0 (0.0)	0.328

*BMI* body mass index, *CAL* clinical attachment level, *CAL5* number of periodontal pockets with CAL ≥ 5 mm, *FMBS* full-mouth gingival bleeding score, *FMPS* full-mouth plaque score, *PISA* periodontal inflamed surface area, *PD* pocket depth, *PD6* number of periodontal pockets with PD ≥ 6 mm.

### Clinical periodontal parameters

Periodontitis was present in 70.4% of responders and 28.6% of non-responders (P = 0.042). Those with good response to OnabotA had worse periodontal condition compared to non-responders (PD: 3.2 ± 0.6 vs. 2.5 ± 0.7 mm, P = 0.006; CAL: 3.8 ± 0.9 vs. 2.9 ± 0.8 mm, P = 0.033; gingival bleeding: 54.1 ± 28.4 vs. 24.4 ± 12.4%, P < 0.001; PISA: 630.0 ± 558.7 vs. 360.1 ± 214.0 mm^2^, P = 0.025) although the levels of plaque accumulation were similar between groups (38.0 ± 22.2 vs. 30.5 ± 23.6%, P = 0.453). In the same line, those participants with better response presented higher number of periodontal pockets with PD ≥ 6 mm and CAL ≥ 5 mm compared to those with worse response to OnabotA (Table 1). Regression analysis showed that diagnosis of periodontitis was linked to good response to OnabotA (OR_unadjusted_ = 5.9; 95% CI 1.0–33.8, P = 0.045). After adjusting for time of evolution, however, the magnitude of this association increased (OR_adjusted_ = 8.9; 95% CI 1.2–61.7, P = 0.026).

### Biomarkers

Responders showed greater levels of inflammation than non-responders (CGRP: 18.8 ± 7.6 vs. 13.0 ± 3.1 pg/mL, P = 0.002; hs-CRP: 3.9 ± 6.6 vs. 0.9 ± 0.8 mg/L, P = 0.003; IL-6: 15.3 ± 8.7 vs. 9.2 ± 4.7 ng/mL, P = 0.016). On contrary, the anti-inflammatory mediator IL-10 was lower in the group of responders than non-responders subjects (2.1 ± 1.1 vs. 3.6 ± 1.1 pg/mL, P = 0.010). When concentrations of inflammatory biomarkers were analysed according to periodontal status, patients with periodontitis presented statistically significant higher levels of IL-6 and CGRP than those without periodontitis (17.0 ± 8.5 vs. 10.0 ± 6.5 ng/mL, P = 0.002 and 19.5 ± 6.9 vs. 15.2 ± 7.8 pg/mL, P = 0.035; respectively) but differences for hs-CRP did not reach statistical significance between groups (3.9 ± 7.0 vs. 2.8 ± 4.8 mg/L, p = 0.465). IL-10 concentrations were statistically significant decreased in the periodontitis group compared to those periodontally healthy (2.0 ± 1.0 vs. 3.0 ± 1.2 pg/mL, P = 0.002).

### Correlation analysis

**Figure 1:**
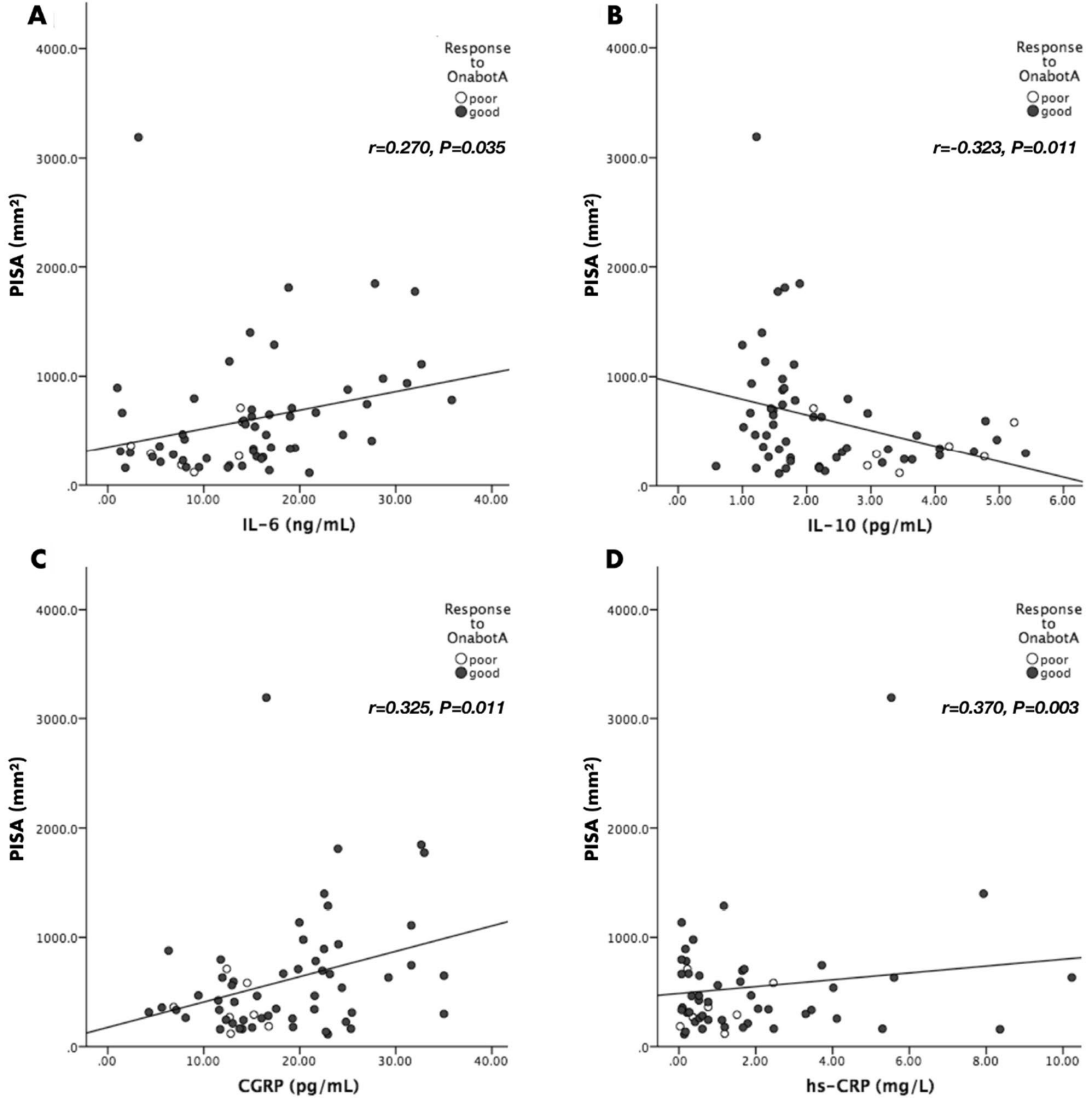
Correlations between PISA (mm^2^) and: (**A**) IL-6 (ng/mL); (**B**) IL-10 (pg/mL); (**C**) CGRP (pg/mL); (**D**) hs-CRP (mg/L).

Correlations between clinical periodontal parameters and biomarkers of inflammation are shown in Table 2. PISA (a measure of active periodontitis) was positively correlated with inflammatory markers whilst the opposite was found with IL-10 (Fig. 1).

**Table 2:** Pearson’s correlation coefficient for clinical periodontal parameters and biomarkers (N = 61).

	PD (mm)	CAL (mm)	FMBS (%)	FMPS (%)	PD6	CAL5
IL-6 (ng/mL)	0.297	0.243	0.464	0.375	0.241	0.387
P-value	**0.020**	0.059	**< 0.001**	**0.003**	0.061	**0.002**
IL-10 (pg/mL)	− 0.406	− 0.455	− 0.234	− 0.186	− 0.378	− 0.404
P-value	**0.001**	**< 0.001**	0.070	0.151	**0.003**	**0.001**
CGRP (pg/mL)	0.313	0.294	0.363	0.311	0.288	0.306
P-value	**0.014**	**0.021**	**0.004**	**0.015**	**0.024**	**0.016**
hs-CRP (mg/L)	0.234	0.215	0.147	0.303	0.145	0.180
P-value	0.070	0.096	0.258	**0.018**	0.267	0.166

*CAL* clinical attachment level, *CAL5* number of periodontal pockets with CAL ≥ 5 mm, *FMBS* full-mouth gingival bleeding score, *FMPS* full-mouth plaque score, *PD* pocket depth, *PD6* number of periodontal pockets with PD ≥ 6 mm.

## Discussion

In this study, we found that in chronic migraineurs with mild systemic inflammation elicited by a common oral infection (i.e., periodontitis) had a better response to OnabotA compared to those without signs of gingival infection. This finding, thus, supports a potential anti-inflammatory effect of OnabotA in the treatment of chronic migraine.

Inflammation is a key element in chronic migraine. CGRP expressed in central and peripheral nervous system modulates nociceptive input and mediates neurogenic inflammation through activation of the trigeminovascular system^23^. CGRP is released inducing vasodilation around cerebral vessels resulting in migraine-like pain^24^ and it has been shown that repeated activation of trigeminovascular system may produce migraine chronification due to central pain sensitization^25^. Besides the role of CGRP as a potential biomarker of chronic migraine^26^, this vasoactive neuropeptide is considered as a predictor of good response to OnabotA^13,14^. Even though the mechanism of action by which OnabotA reduces the number and severity of headaches in chronic migraineurs is not fully understood, it might involve the blockage of the release of neuropeptides and other inflammatory mediators in sensory neurons that promote peripheral sensitization within trigeminal glia^27^. Additionally, at the level of the spinal cord OnabotA could inhibit release of pro-inflammatory mediators which results in deactivation of second-order nociceptive neurons and glia cells involved in central sensitization^27^. Contradictory to these hypotheses, evidence from different animal models did not find an association between reduction of pain and inflammation after OnabotA injection, thus, questioning the anti-inflammatory effect of this treatment modality^8^.

Periodontitis is able to induce a systemic inflammatory response with high levels of pro-inflammatory cytokines and acute-phase reactants measured in the peripheral blood^2^. A recent case–control study showed that subjects with periodontitis had two-fold increased risk for having chronic migraine^15^. On top of that, another clinical study demonstrated that periodontal inflammation was associated with increased circulating levels of CGRP in chronic migraineurs and authors hypothesized that upregulation of IL-6 could mediate this finding^3^. However, whether IL-6 induces CGRP overexpression or vice versa is still unknown as CGRP is capable of producing inflammatory cytokines from lymphocytes and macrophages after lipopolysaccharide infection^28,29^. In the present study, different periodontal parameters reflecting active disease (i.e., PD and PISA) were positively correlated with elevated levels of IL-6 and CGRP.

For the purpose of this study, we used a human model that mimics a sufficient mild systemic inflammatory response that allowed us to confirm the anti-inflammatory effect of OnabotA in chronic migraine as those subjects with untreated periodontitis and increased concentrations of CGRP and IL-6 responded better to this therapy than those without periodontitis. We have to be very cautious, however, when interpreting these results. Periodontitis is an oral infection that has to be treated; otherwise, it would lead to tooth loss and diminished masticatory function. Also, it has a systemic impact increasing the risk not only of chronic migraine but also of other conditions such as cardiovascular/cerebrovascular diseases, diabetes, dementia, kidney disease, and rheumatic diseases among others^30^. In our study, after periodontal examination those with a diagnosis of periodontitis were immediately offered periodontal treatment or were recommended seeking for treatment. What is unknown is whether the effect observed in our study is maintained after evaluation of efficacy at 3 months. Another question to be answered would be if periodontal therapy could have an impact on migraine outcomes or OnabotA efficacy.

We have to acknowledge some limitations in relation to this investigation. Firstly, blood inflammatory markers were determined only prior to OnabotA injections. Future studies might include a post-treatment blood sample collection to assess whether these biomarkers are reduced or not as previously shown for CGRP^12^. Secondly, although in apparent good general health, some of the patients could have other undiagnosed conditions linked to increased systemic inflammation. For instance, responders had higher BMI than non-responders and it is well-known that increased BMI often co-exists with low-grade chronic inflammation^31^. Nevertheless, only two participants from our study (one from each study group) presented a BMI value ≥ 30 kg/m^2^ and could be considered as obese. Thirdly, study sample size is small in particular the number of non-responder was 7. This is because patients were recruited from a Headache Unit with experience treating chronic migraine subjects and most of them were good responders. Future trials with a formal sample size calculation including similar number of patients in each group are warranted. Another potential limitation could be the follow-up to assess treatment response in our patients (24 weeks after first injection). Although this follow-up is in accordance with the PREEMPT protocol^17^, which measures the primary outcome at the same time, because after third injections patients could still show some improvement^32^, further evidence is needed to confirm our findings in the long-term (e.g., 56 weeks after first injection).

To conclude, the present data show that in presence of elevated systemic inflammatory markers related to periodontitis, OnabotA seems to reduce migraine attacks. The changes of scheduled inflammatory parameters after treatment and for an adequate period must be done.

## Data Availability

The dataset analysed during the current study are available from corresponding author on reasonable request.
